# Urban exodus? Understanding human mobility in Britain during the COVID‐19 pandemic using Meta‐Facebook data

**DOI:** 10.1002/psp.2637

**Published:** 2022-12-07

**Authors:** Francisco Rowe, Alessia Calafiore, Daniel Arribas‐Bel, Krasen Samardzhiev, Martin Fleischmann

**Affiliations:** ^1^ Department of Geography and Planning University of Liverpool Liverpool UK; ^2^ Edinburgh College of Art University of Edinburgh Edinburgh Scotland UK; ^3^ The Alan Turing Institute British Library London England UK

**Keywords:** COVID‐19, Great Britain, human mobility, internal migration, Meta‐Facebook, mobile phone location data, urban exodus

## Abstract

Existing empirical work has focused on assessing the effectiveness of nonpharmaceutical interventions on human mobility to contain the spread of COVID‐19. Less is known about the ways in which the COVID‐19 pandemic has reshaped the spatial patterns of population movement within countries. Anecdotal evidence of an urban exodus from large cities to rural areas emerged during early phases of the pandemic across western societies. Yet, these claims have not been empirically assessed. Traditional data sources, such as censuses offer coarse temporal frequency to analyse population movement over infrequent time intervals. Drawing on a data set of 21 million observations from Meta‐Facebook users, we aim to analyse the extent and evolution of changes in the spatial patterns of population movement across the rural–urban continuum in Britain over an 18‐month period from March 2020 to August 2021. Our findings show an overall and sustained decline in population movement during periods of high stringency measures, with the most densely populated areas reporting the largest reductions. During these periods, we also find evidence of higher‐than‐average mobility from high‐density population areas to low‐density areas, lending some support to claims of large‐scale population movements from large cities. Yet, we show that these trends were temporary. Overall mobility levels trended back to precoronavirus levels after the easing of nonpharmaceutical interventions. Following these interventions, we found a reduction in movement to low‐density areas and a rise in mobility to high‐density agglomerations. Overall, these findings reveal that while COVID‐19 generated shock waves leading to temporary changes in the patterns of population movement in Britain, the resulting vibrations have not significantly reshaped the prevalent structures in the national pattern of population movement. As of 2021, internal population movements sit at an intermediate level between those observed pre‐ and early phases of the pandemic.

## 1 INTRODUCTION

The COVID‐19 pandemic has led to major changes in the patterns of human mobility within and between countries. In addition to stringent international travel restrictions, nonpharmaceutical interventions to contain the spread of COVID‐19 have transformed daily life behaviours resulting in reduced overall levels of mobility (Nouvellet et al., 2021). Especially during lockdowns, mobility recorded reductions in the frequency, distance and time of trips across the world (e.g., Department for Transport, 2021). Rises in teleworking, online schooling and remote shopping activity reduced the need to travel for work, education, shopping and leisure. Coupled with fears of crowded public spaces, nonpharmaceutical interventions prompted more geographically localised mobility patterns (Engle et al., 2020; Linka et al., 2021), and modal transport shifts from mass public transit to private, active and e‐forms of mobility (Li et al., 2021).

These changes and fears generated a passionate debate about the future of big cities during early stages of the pandemic. Some predicted that COVID‐ 19 would create a tipping point leading to ‘the end of cities’, while others made predictions anticipating strong urban recovery and resilience (e.g., Florida et al., 2021). Building on existing knowledge, studies have carefully analysed existing evidence and anticipated the potential immediate and long‐term economic and social reverberations of the COVID‐19 pandemic on the structure and morphology of cities and regions (Sharifi & Khavarian‐Garmsir, 2020). These changes are anticipated at a microgeographical scale, altering the organisation of people and activity within urban regions at fine granular spaces, such as neighbourhoods (Florida et al., 2021). Yet, these changes are not expected to significantly reshape existing structural national patterns of population settlement and economic systems at a macrogeographical scale (Florida et al., 2021).

However, little is known about the patterns of population redistribution across the national territory during the COVID‐19 pandemic. Recent empirical evidence has documented an increase in internal migration from large cities to rural areas in Australia (Borsellino et al., 2022), Germany (Stawarz et al., 2022), Japan (Fielding & Ishikawa, 2021), Spain (González‐Leonardo & Rowe, 2022; González‐Leonardo et al., 2022) and Sweden (Vogiazides & Kawalerowicz, 2022). However, internal migration captures movement over a fixed interval, missing short‐term residential moves. Capturing both short‐ and long‐term residential moves, as well as regular forms of mobility (such as commuting), is key to understand their interactions and determine the durability of population movement during the progression of COVID‐19.

A key challenge to capture national‐scale patterns of population movements across the rural–urban continuum as the pandemic evolves has been the lack of suitable data. Traditionally census and population register data have been used to explore human mobility patterns at such scale. However, these data systems are not regularly updated, lacking the temporal granularity to analyse population movements over short‐time intervals. Digital traces data derived from mobile phone applications now provide a unique opportunity to capture these movements at an unprecedented spatial and temporal granularity (Green et al., 2021).

Drawing on Meta‐Facebook users' mobile phone location data, this paper aims to analyse the extent and durability of changes in human mobility patterns across the rural–urban continuum in Britain during the COVID‐19 pandemic, covering an 18‐month period from March 2020 to August 2021. Specifically, this paper seeks to address the following set of questions:

- To what extent have people moved away from cities, and redistributed across the urban–rural continuum during the pandemic?
- What have been the key interactions between places across the population density hierarchy? Have people mainly moved to sparely populated areas?
- To what extent the intensity of population movement from cities have been sustained throughout the pandemic? Have the observed changes been temporary, or are likely to persist postpandemic?

Our work contributes to expanding existing knowledge by offering new empirical evidence on the impacts of COVID‐19 on population movement. First, it provides a systemic understanding of the overall population movement system within Britain. While prior work has focused on discrete forms of human mobility, our study provides an overall understanding of the ways various forms of human mobility have evolved during COVID‐19. Second, we capture the evolution of the internal population movements during 2020 and 2021. Data limitations have prevented previous studies from extending their analysis to understand the patterns of internal migration beyond 2020. Third, we contribute some of the first empirical evidence documenting the patterns of internal population movement across the urban–rural continuum.

The rest of the paper is structured as follows. The next section reviews the emerging evidence and hypotheses about the spatial patterns of population movements from cities during the COVID‐19 pandemic, before discussing the predominant trends of population movements in Britain in the years preceding the pandemic. Section 3 describes the data, and Section 4 discusses the methods used in this study. Section 5 presents the key results from our analyses before they are discussed in light of the existing literature in Section 6, which also identifies key limitations and potential avenues for future work.

## 2 BACKGROUND

### 2.1 Emerging evidence on mobility patterns across the urban hierarchy during COVID‐19

As COVID‐19 expanded throughout the world in early 2020, anecdotal evidence of an ‘urban exodus’ from big cities emerged in many western societies (Sagnard, 2021; Weeden, 2020). At the early stages of the pandemic, little was known about the virus, and globally connected cities were hit hardest (Florida et al., 2021; Matheson et al., 2020). By November 2020, approximately 95% of all the reported infections and fatalities had occurred in a few large cities (Pomeroy & Chainey, 2020). Newspapers' headlines were speculating about ‘the end of cities’ (e.g., Pomeroy & Chainey, 2020). In the United Kingdom, reports indicated that the number of online inquiries from residents living in the 10 largest cities looking for a village property was reported to increase by 126% in June–July 2020, relative to the same period in 2019 (Marsh, 2020). In France, increases in real estate transactions outside cities were also linked to city residents moving to smaller towns or villages (Sagnard, 2021). In the United States, a rise of 30 percentage points in the number of households moving from large metropolitan areas was reported based on data from mail‐forwarding requests and credit report data (Paybarah et al., 2020).

COVID‐19 exposed key imperfections of living in large cities and these were used to articulate the ‘urban exodus’ narrative. Facilitated by high air‐travel connectivity, job density and spatial concentration of public‐facing jobs, large cities became early global epicentres of COVID‐19 infections during the early stages of the pandemic (Florida et al., 2021). Precoronavirus housing affordability and poor housing conditions were consistently identified as urban challenges in large cities. Coupled to these issues, lockdowns, social distancing, remote work and homeschooling augmented the pressure for families living in small and crowded living spaces, to move out of cities in the look for more space and affordable housing (Hernández‐Morales et al., 2020; Hughes, 2020). Teleworking, increased familiarity and the use of online shopping reduced the need for commuting and living in proximity to work and retail locations. Business closures removed the effervescence of urban entertainment, leisure and social spaces and triggered a rapid spike in unemployment in many countries as nonesential, public‐facing work was suddenly paused during 2020 (e.g., Falk, 2020).

Enabled by automation and artificial intelligence, new digital technologies have greatly facilitated the transition to remote activities and arguably away from large cities during COVID‐19 (Ting et al., 2020). Technologies, such as video conferencing, shared documents, instant messaging and cloud computing became instrumental in enabling remote work and education (Al‐Maroof et al., 2020; Vargo et al., 2021). Virtual services, like video streaming and social media platforms offer access to some of the cultural effervescence and community that have consistently been an important factor drawing people to large cities (Glaeser et al., 2001). Online shopping platforms, such as Amazon and Ebay now provide an opportunity to buy and ship products from distant locations (Ting et al., 2020).

However, preliminary evidence suggests that population movements during the pandemic have been over relatively short distances. Evidence from the United States and Spain suggests that most of the movement from large cities during the pandemic has been to their suburbs, as opposed to smaller, remote cities and towns (González‐Leonardo, Rowe, et al., 2022; Hughes, 2020). Yet, some city leavers also appear to have moved to neighbouring areas, second residences, holiday destinations and other cities (Kolko et al., 2021; Paybarah et al., 2020). In Australia, larger cities have been the primary destination for migration from other large cities, while the flow of people moving down the urban hierarchy has been much smaller (Davies, 2021). Preferences seem to have changed during the pandemic with people looking for green natural environments in rural locations (Borsellino et al., 2022). COVID‐19 restrictions also seemed to have resulted in a substitution effect in some countries. In Britain, while COVID‐19 restrictions were generally used to discourage mobility, people were allowed to perform some outdoor activities in their local area, even during periods of high stringency. As a result, while overall mobility is suspected to have declined, local levels of mobility are expected to have increased during early stages of the pandemic.

Persuasive cases have been made against headlines speculating about the end of cities. Past pandemics wreaked havoc and substantially influenced medical, cultural, political and urban design changes, but they have not dented the key role that cities play in society (Glaeser, 2020). For instance, the Black Plagues of the 14th century killed one‐third of the population in Europe and the Middle East (Pamuk, 2007). The Cholera outbreaks of the 19th century decimated large cities across the world, including London, Paris, Moscow, Hamburg, New York and Madrid (Ali et al., 2015; Briggs, 1961). Yet, large cities have continued to be important gravitational centres for population concentration.

Cities are critical engines of innovation, economic growth and prosperity. They enable the emergence of agglomeration economies. Concentration in cities facilitates the exchange of goods, knowledge, information and ideas by reducing transportation and communication costs, offering abundant critical mass and fostering strong firm linkages (Glaeser, 2010). A fundamental ingredient underlying these benefits is the face‐to‐face interaction that can be fostered by urban agglomerations (Storper & Venables, 2004). While routine, codified activities can be more easily communicated and performed virtually from remote locations, complex, innovative and less familiar tacit knowledge, tasks and ideas require face‐to‐face contact (Storper & Venables, 2004). This need for face‐to‐face interaction comprises an essential reason why the proliferation of Internet communication has not led to the spatial diffusion of urban agglomerations and ‘the death of distance’, despite its capacity to enable complex ways of communication between distant locations (Fujita & Thisse, 1996).

Additionally, rural and remote areas may lack the infrastructure and services needed to support incoming urban residents. These areas do not offer the vibrancy and sophistication of entertainment, cultural and convenient services that urbanities are used to. Telework is likely to remain a permanent way of interaction postpandemic. Yet, poor broadband connectivity in rural and remote locations has remained a key challenge across most countries in the world (OECD, 2020). Not all forms of work can be done remotely, including: high‐touch, public‐facing work providing essential (e.g., healthcare and education services) and nonessential (e.g., restaurants and bars) services; essential, nonpublic facing work related to construction, infrastructure and maintenance and knowledge‐intensive activities requiring high‐level abstraction and cognitive capacity (i.e., teaching) (Florida et al., 2021). Also online work fatigue has become a new phenomenon, which has been coined ‘Zoom fatigue’ (Fosslien & Duffy, 2020). Rather than full‐time remote work, hybrid forms of work are more likely to outlast the pandemic, requiring flexibility to combine office and online presence. Such change may entail a need for reliable broadband connectivity and accessibility to employment centres.

Thus, while speculations during early stages of the pandemic pointed to an ‘urban exodus’ as COVID‐19 cases and deaths surged in large cities, emerging evidence suggests that the effects from the pandemic have reverberated through to the internal mobility system of countries across the world prompting residential relocations from large cities. Yet, such shocks are less likely to have led to a significant reconfiguration of the national mobility system. Rather, they may have accelerated existing mobility trends, with cities expected to bounce back and remain major centres of population attraction postpandemic. Thus far, however, existing evidence remains largely anecdotal. We seek to offer some first evidence assessing the ways in which the British mobility system has weathered from the beginning of the pandemic to the reopening of the country's economy. Specifically, we seek to offer evidence of (1) relatively high local mobility; (2) high intensity of mobility from high‐density city areas to less dense areas (including intermediate‐density areas, e.g., suburbs and medium‐size cities); (3) high intensity of mobility between high‐density city areas to low‐density areas (including sparsely populated, rural areas and coastal towns) during early stages of the pandemic and (4) a decrease in the intensity of mobility from highly dense city areas as COVID‐19 stringency eased.

### 2.2 Contemporary mobility patterns across the British urban hierarchy

To determine the extent of change in mobility patterns during COVID‐19, we review preexisting predominant trends in the British mobility system. Globally, the United Kingdom occupies an intermediate rank in terms of overall levels of mobility. Estimates based on 2011 census data, indicate that 6.8 million individuals changed their usual residential address in the last 12 months (Rowe et al., 2020). That is above the global average but well below highly mobile countries (Bell et al., 2015). In the United Kingdom, while the share of short‐distance migratory moves has been declining, most movement still occurs locally with approximately 30% of all residential changes taking place within 10 km. Less than 7% happen between distances of 50–200 km and less 3% exceed 200 km (Champion & Shuttleworth, 2017). These patterns reflect the prevalence of suburbanisation across British cities (Bailey & Milton, 2018).

A historical feature of the internal population movement in the United Kingdom for over the last half‐century has been counterurbanisation (Champion, 1989). This process is characterised by population losses due to internal population movement in major metropolitan areas and gains in smaller towns and rural areas. Over the last decade, a general decline in the size of net migration gains and loses across the national system has resulted in a weakening of the counterurbanisation process (Lomax et al., 2014), resulting in minimal population redistribution across the national urban settlement (Rowe et al., 2019). Acute net migration losses have concentrated in London, the urban conurbation of the West Midlands, metropolitan districts in the North West, Glasgow, Edinburgh and Belfast (Lomax et al., 2014). Primary areas of net gain have been districts in the South West, the south coast and in the East of England (Lomax et al., 2014). Since 2008 following the global financial crisis, metro‐to‐metro moves have replaced metro‐to‐nonmetro moves as the predominant direction of migration flows in the United Kingdom (Lomax & Stillwell, 2017). Nonmetro‐to‐metro moves have increased exceeding nonmetro‐to‐nonmetro moves, although both of these types of moves have remained smaller than those occurring between metro areas, and from metro to nonmetro locales (Lomax & Stillwell, 2017).

Student mobility is also a key feature of the internal population movements in Britain. Over 75% of people aged between 15 and 24 changed residential address between 2001 and 2011; that is, three in four people (Champion & Shuttleworth, 2017). While this share has recorded a steady declining trend since 1971, young people continue to be among the most mobile groups in Britain (Champion & Shuttleworth, 2017). Students tend to move an average distance of 100 km from their parental home to attend university. Most of these moves are of temporary nature, linked to 6‐ or 12‐month tenancy agreements, drawing disproportionate numbers from sparsely populated areas (Faggian & Mccann, 2009). During the early stages of the COVID‐19 outbreak, the closure of universities and social distancing restrictions are likely to have prompted large flows of students out of large cities back to their parental homes.

The importance of London in redistributing the population represents an additional feature of the national population mobility system. Between 2010 and 2011, moves from and to London accounted for 15% of the total migration moves between local authority districts in the United Kingdom (Lomax & Stillwell, 2017). London plays a key role as a social escalator region attracting young adults at rates which are higher than elsewhere in the country but recording significant losses of the population due to migration across all other age groups as they step off the escalator and move away from London (Fielding, 1992). London has consistently registered net migration losses, although these losses have lessened during the 2000s as a result of less acute outflows and greater inflows, particularly in inner boroughs (Champion, 2015). Over the last four decades, London has registered the largest net migration losses during periods of national prosperity and lowest negative balances when economic conditions have been less buoyant (Lomax & Stillwell, 2017). This evidence suggests more severe migration losses in London during the COVID‐19 pandemic, given a surge in unemployment and benefit claims. Next, we describe the data and methods used to assess the extent to which the existing patterns of population movement have been altered during the COVID‐19 pandemic.

## 3 DATA

To capture population movements during the COVID‐19 pandemic, we used anonymised aggregate mobile phone location data from Meta‐Facebook users comprising 21 million observations for Great Britain, covering an 18‐month period from 23 March 2020 to 15 August 2021. We used two data sets Facebook Movements and Facebook Population created by Meta and accessed through their Data for Good Initiative (https://dataforgood.facebook.com). The data sets are built from information from users who shared their location history. Before sharing the data, Meta applies three techniques to ensure privacy and anonymisation: Random noise, spatial smoothing and dropping small counts. First, a small undisclosed amount of random noise is added to ensure that precise location cannot be identified for small population counts in sparsely populated areas. While removing small counts may underrepresent the population in these places, it also reflects the actual geographic pattern of population distribution. Second, spatial smoothing is applied to produce a smooth population count surface using inverse distance‐weighted averaging. Third, any remaining population counts of less than 10 are removed from the final data set—see Maas et al. (2019) for details.

The Facebook Movements data set provides information on the total number of Facebook users moving between and within locations in the form of origin‐destination matrices. The Facebook Population data set provides information on the number of active Facebook users in a location at a given point in time. Both data sets provide daily aggregate population counts over three windows of 8 h: 00:00–08:00, 08:00–16:00 and 16:00–00:00, which are used to define the location of users. Meta defines the location of users as the place where they spent most of their time at a given time window (e.g., 00:00–08:00). Comparing the location of individuals between two temporal windows provides data on the number of people moving between locations. Both data sets include a baseline population count indicating the number of Facebook users moving between locations, or a total number of Facebook users in a given location during a fixed baseline period. The baseline period is defined as an average of the population counts covering 45 days; that is, the 45 days before 10 March 2020. The data sets also include a ‘quality’ score indicating the number of standard deviations by which the observed population count at a given time point differs from the baseline population count, highlighting statistically significant changes in population counts. We note that the aggregate nature of the data does not allow distinguishing between various forms of population movement, for example, permanent and daily population moves. However, we distinguished between movements between areas and movements within an area, to capture differences in the impact of the pandemic on mobility. As described above, while COVID‐19 restrictions are expected to have discouraged long‐distance movements (i.e., between areas), they are argued to have produced increased levels of local mobility (i.e., within an area).

Facebook used the Bing Maps Tile System developed by Microsoft as a spatial reference framework to organise the data. It is a geospatial indexing system that partitions the world into tile cells in a hierarchical way, comprising 23 different levels of detail (Schwartz, 2018). At the lowest level of detail (Level 1), the world is divided into four tiles with a coarse spatial resolution. At each successive level, the resolution increases by a factor of two. The Facebook mobility data we used are based on tiles at level of detail 12, which provides ground metres‐to‐pixel resolution of 38.2185 measured at the Equator. For Britain, that is a tile size of approximately 5.5–6 km^2^.

We also used 1 km^2^ gridded population data produced by Patias et al. (2019) derived from the 2011 UK Census to analyse population movement across the rural–urban continuum. We used gridded resident population counts to ensure consistency with the Meta‐Facebook data. Patias et al. (2019) derived gridded population data from British censuses by calculating the correspondence between small area census geographies and 1 km^2^ grids, and allocating population counts from each census area unit to its conforming 1 km^2^ grids. We aggregated these data to Bing tile level 12 to match the Meta‐Facebook data.

We used the resulting data to estimate population density at the Bing tile level over the rural–urban continuum in Britain. This allows overcoming issues of comparability, spatial scale and measurement associated with the use of binary rural/urban classifications (Fielding, 1989). We used deciles of population density to classify Bing tiles into 10 discrete categories. Figure maps the resulting population density classes, which tend to correspond to the Office for National Statistics (ONS) rural/urban classification (see Supporting Information: Figure 1). We preferred our population density classification as it provides a consistent definition of areas based on population density. The ONS rural/urban classifications for England, Wales and Scotland are generated independently based on different input data, and definitions of rural and urban—see Supporting Information: Figure 2.

**Figure 1:**
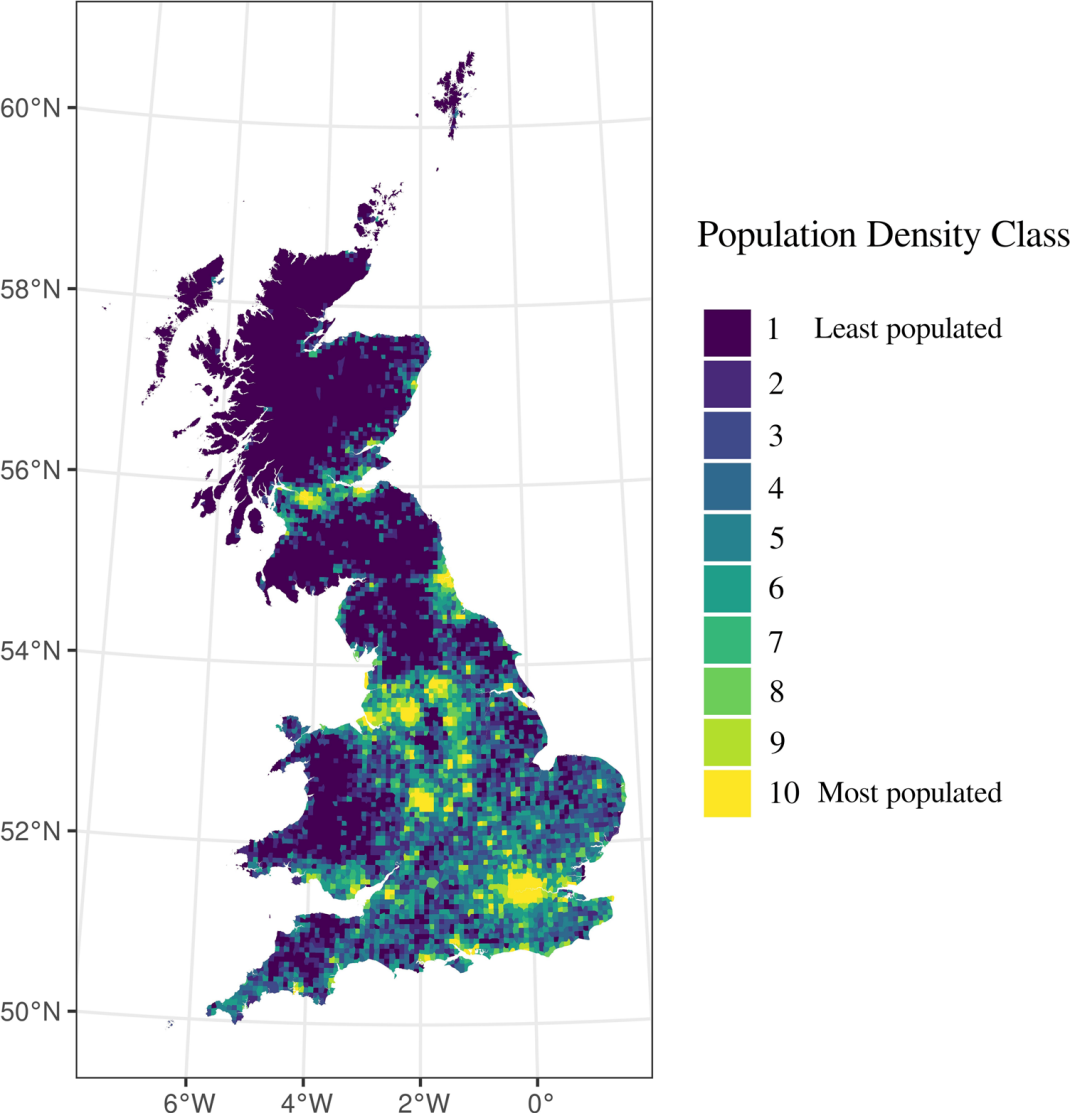
Spatial distribution of Facebook tiles into population density classes. Class 1 includes the least densely populated, representing sparsely populated rural areas. Class 10 includes the most densely populated and highly urban agglomerations.

**Figure 2:**
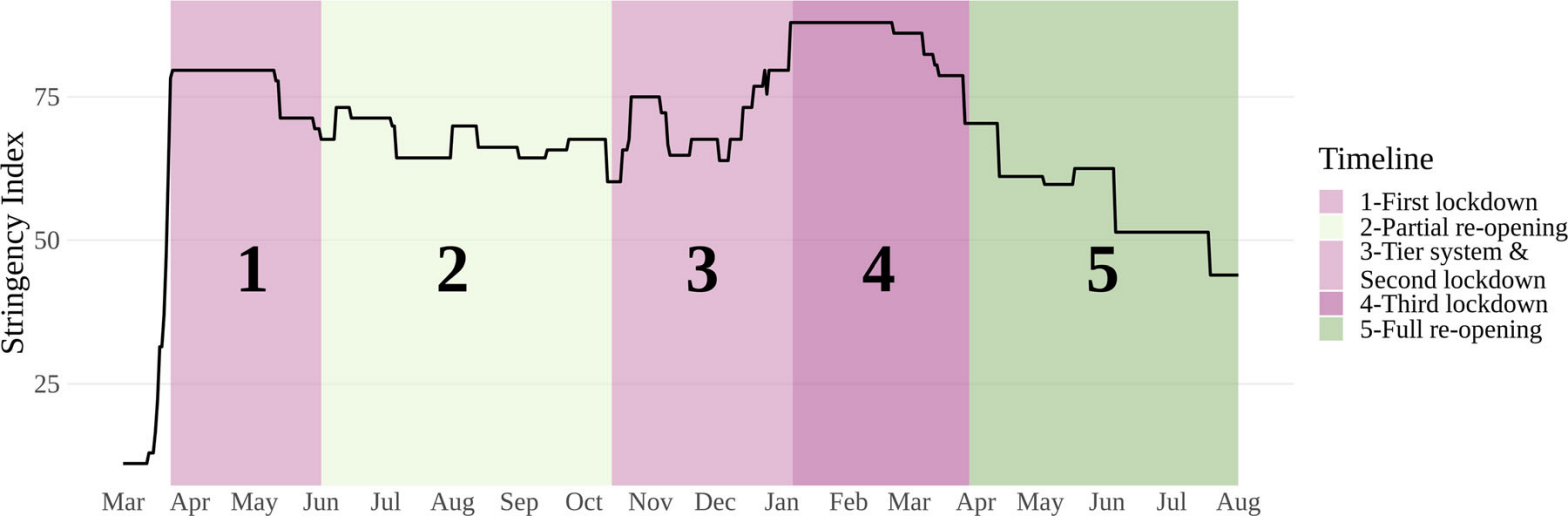
Level of COVID‐19 stringency measures in Britain, March 2020 to August 2021. The stringency index measures the level of nonpharmaceutical interventions to COVID‐19, such as social distancing and lockdown measures and ranges from 0 to 100 (100 = strictest). The stringency index was sourced from the COVID‐19 government response tracker (https://www.bsg.ox.ac.uk/research/research-projects/covid-19-government-response-tracker)—see Hale et al. (2020) for more information.

## 4 METHODS

The analysis involved two steps. First, we used area‐based mobility metrics to measure the extent of change in mobility inflows, outflows and intraflows across the urban hierarchy at two key discrete points during the COVID‐19 pandemic in Britain (i.e., after the first lockdown and after the implementation of the reopening). A description of the timeline of the COVID‐19 pandemic in the United Kingdom is provided in Section 5.1. Second, we used statistical modelling to assess spatial and temporal variations in the intensity of mobility flows across specific origin‐destination pairs of population density classes over the course of the pandemic. We adopted an open and reproducible research approach and produced an open data product (Arribas‐Bel et al., 2021), including reproducible computational code to reproduce or extend our analysis, see details in the Data Availability Statement.

### 4.1 Area‐based mobility metrics

We measured changes in mobility inflows, outflows and intraflows across the urban hierarchy between two distinctive periods during the course of the COVID‐19 pandemic: (1) after the announcement of the first lockdown between 23 March 2020 and 19 April 2020, and (2) after the implementation of the government's reopening plan out of lockdown between 19 July 2021 and 16 August 2021. We measured changes in mobility during these periods, relative to precoronavirus levels during the baseline period as defined by Meta‐Facebook (i.e., a period covering 45 days before 10 March 2020). Specifically, we computed the percentage change in mobility inflows, outflows and intraflows by population density class as follows:

(1) $$I_{\mathrm{tc}}=\left(\frac{{\tilde{x}}_{\mathrm{tc}}}{{\tilde{b}}_{c}}-1\right)\;\times \;100,$$

where $\tilde{x}$ corresponds to the median count for a specific type of population movement (i.e., inflows, outflows or intraflows); b is the baseline median mobility count; t relates to the two periods described above; c refers to each population density class as defined in Section 3. A positive I score indicates an increase in the extent of population movement relative to the baseline prepandemic period. A negative I score represents a decrease, while a zero score denotes no changes.

### 4.2 Modelling tile‐to‐tile mobility flows

In the second stage, we used statistical modelling to understand differences in the intensity of mobility between tiles of different population density classes (as presented in Figure 1) and the extent to which the intensity of movement has evolved over time as the pandemic unfolded. In principle, the overall number of people moving between tiles of different population density classes could be estimated based on the original raw data. However, this approach could be misleading. Movement flows are not only influenced by the population nature of the origin and destination areas. Distance between locations, size, or socioeconomic characteristics, among others, may also play a role as confounders, masking the true effect of population class. To unpick each of these and derive a ‘cleaner’ estimate of the relevance of the nature of population, we opted for a regression modelling approach.

We adopted a spatial interaction framework (Rowe, Lovelace, et al., 2022). We used population flows between tiles by time window and day (as described in Section 3), and modelled them as a function of characteristics of the flow itself (*F*_*ij*_), the origin (*Tile*_*i*_) and destination (*Tile*_*j*_) tiles and the temporal nature of the flow (*T*_*w*_). Crucially, we included indicator variables that capture the pair of population classes (Figure 1) of the origin and destination tile. In mathematical form:

(2) $$\mu_{ijw}=\alpha +\underset{F_{ij}}{\underbrace{\sum_{IJ}\gamma_{IJ}+\beta_{1}d_{ij}+\beta_{2}q_{ijw}}}+\overset{Tile_{i}}{\overbrace{\beta_{3}Pop_{i}}}+\underset{Tile_{j}}{\underbrace{\beta_{4}Pop_{j}}}+\overset{T_{w}}{\overbrace{D+H+Wk}},$$

where *µ*_*ijw*_ is the expectation of the flow of people from tile *i* to tile *j* in the time window *w*; *⍺* is an intercept; $\gamma_{\mathrm{IJ}}$ is a series of indicator variables that reflect the pair of population density classes of a given origin *i* (*I*) and destination tile *j* (*J*), resulting in 99 pairs (10 classes × 10 classes minus one so it is not collinear with *⍺*); *d*_*ij*_ is the geographic distance between tiles *i* and *j*; *q*_*ijw*_ is a measure of the quality of the flow estimate provided by Meta‐Facebook and related to the uncertainty behind the user count of the flow as described in Section 3; *Pop*_*i,j*_ represents the population at the origin (*i*) and destination (*j*) tiles; $\beta_{1,2,3,4}$ are parameters to estimate in the model linking their respective covariates to *µ*_*ijw*_; *D* is a trend tracking the day to which the flow relates to during the period in analysis; while *Wk* and *H* are indicator variables capturing day of the week (i.e., weekday or weekend) and hour window (i.e., 00:00–08:00, 08:00–16:00 and 16:00–00:00).

Our focus in Equation (2) is centred on $\gamma_{\mathrm{IJ}}$. Controlling for all other variables, these parameters capture the extent to which, the expected flow between a given origin‐destination population density class pair of tiles (e.g., a high‐density origin to a low‐density destination) is systematically higher or lower than if it occurred between a baseline origin‐destination population density class pair of tiles (e.g., a low‐density origin to a low‐density destination). Additionally, we standardised continuous variables (*d*_*ij*_, *q*_*ijw*_, *Pop*_*i j*_), *α* so that they can be interpreted as the expected flow on the first day (*D* = 0), during the first temporal window (*H* = 00:00–08:00), on a weekday (*Wk* = 0), for the baseline origin‐population population density class pair, when all the other variables are at their mean value. In this context, each $\gamma_{\mathrm{IJ}}$ can also be seen as the ‘modulation factor’ around that expectation associated with each pair of origin‐destination classes. The baseline origin‐destination population density class pair is the lowest population density class as origin and destination.

We used a count data regression model. Specifically, we fitted a generalised linear model (GLM) where the error term is assumed to be distributed following a Poisson distribution, with a flow expectation of (*µ*_*ijw*_) linked to the flow count (*F*_*ijw*_) through a log link:

(3) $$\log\;E(F_{ijw})=\mu_{ijw},$$

(4) $$F_{ijw}\sim Pois(e^{\mu_{ijw}}).$$

The Poisson regression model (PRM) assumes that equidispersion, that is, equality of mean and variance in the response variable (Cameron & Trivedi, 2013). In practice, the equidispersion property is commonly violated because of overdispersion, that is, the variance exceeds the mean. When this occurs, the PRM may produce biased parameter estimates, causing the standard errors of the estimates to be underestimated, and compromising the statistical inference process (Hilbe, 2011). To test for overdispersion, we used a regression‐based test based on an auxiliary regression of the conditional variance as described in Cameron and Trivedi (2013).

Following Gelman and Hill (2006), we used a quasi‐PRM to address overdispersion in our response variable. This is one of the most common strategies to deal with overdispersion in count data models (Hilbe, 2011). Intuitively, this model adjusts the standard errors of the estimates to account for the extra dispersion in the data. To implement this, we estimated Equation (2) by using robust variance estimators. The number of active Facebook users were used as a weight to account for the variability of the observed count of population movement over time. This strategy is also used to mitigate for any potential biases regarding the variation in the observed number of active Facebook users changes over time across Britain.

We fitted Equation (2) using iteratively reweighted least squares (IWLS). We separately estimated models for individual months in our data, resulting in 18 sets of estimates. Our key aim was to generate estimates for *⍺* and $\gamma_{\mathrm{IJ}}$, so that we focused on discussing the evolution of these estimates in a grid of line plots with 10 rows and 10 columns, each of them representing one of our population density classes. The plot corresponding to the *I*th row and *J*th column displays the evolution of the parameter that tracks the intensity of population flows from tiles in population density class *I*th to those in population density class *J*th.

## 5 RESULTS

### 5.1 Timeline of COVID‐19 in Britain

Understanding the timeline of government interventions during the COVID‐19 pandemic in Britain is critical to the broader context within in which mobility patterns have occurred. A range of nonpharmaceutical interventions was implemented to reduce the spread of COVID‐19 during the pandemic. Figure 2 shows the stringency index for the 18 months period covered by our data (March 2020 to August 2021). The stringency index is a composite measure developed by Hale et al. (2020), capturing the level of restrictions imposed by governments, ranging from 0 (loosest) to 100 (strictest).

Figure 2 identifies key five time intervals, with pink indicating increases in stringency and green denoting reductions in stringency. Darker colours point to large changes. The first period corresponds to the first lockdown, which was announced on 23 March 2020 and imposed a ‘stay at home’ order. During this period, only essential travel and outdoor physical activities were permitted. The second phase involves a period of partial reopening, with schools and nonessential shops being reopen on 1 June 2020. Workers who could not work from home were permitted to return to workplaces, but public transit was to be avoided. This partial reopening was reflected in moderately high stringency levels. The third phase involves a period of fluctuating levels of stringency between 14 October 2020 until 6 January 2021 in response to changing COVID‐19 cases and hospitalisation rates. It was marked by the introduction of a tier system with local lockdowns to locally contain centres of contagion, and then followed by a second national lockdown between 5 November 2020 and 2 December 2020. The fourth phase was characterised by a third national lockdown announced on 6 January 2021 to contain a rapid rise in COVID‐19 cases due to the Delta variant. Levels of stringency during this phase were the strictest. The fifth and final phase represented in Figure 2 displays the implementation of the government's plan out of the third national lockdown, which sought to gradually lift existing restrictions in four steps. Step 1 began on 8 March 2021 with the reopening of schools and outdoor gatherings, followed by nonessential shops and outdoor venues (Step 2), and subsequently indoor venues reopening and large outdoor events (Step 3). The reopening strategy was completed on ‘freedom’ day on 19 July when most of COVID‐19‐related legal restrictions were lifted.

### 5.2 Mobility patterns after the first lockdown and ‘freedom’ day

**Figure 3:**
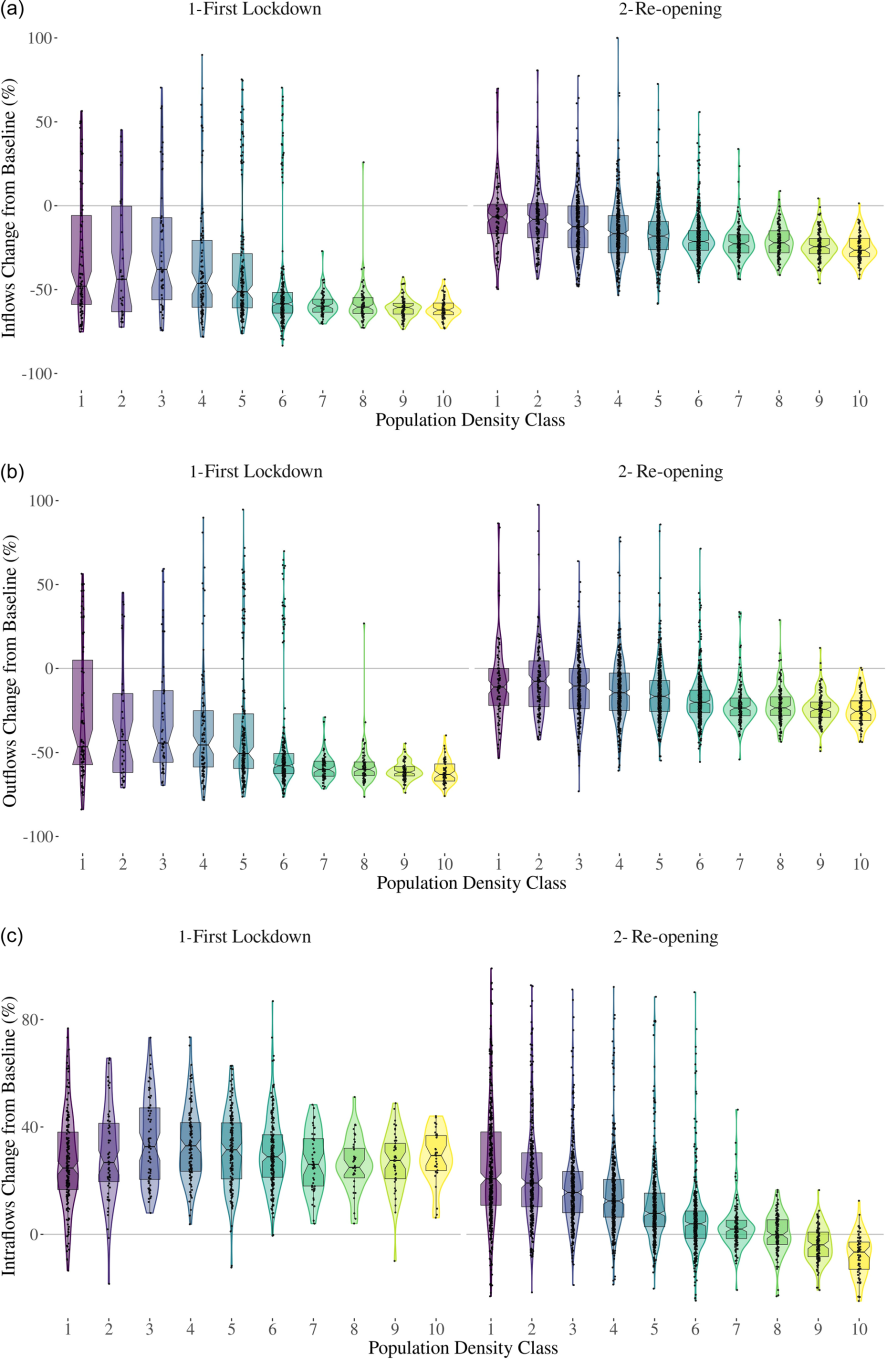
Inflows (a), outflows (b) and intraflows (c) percentage change from prepandemic mobility standards after the first lockdown and ‘freedom’ day across different population density classes. Positive values indicate % increase in flows compared to the baseline, negative values indicate % decrease, while zeros signal no change.

Next we analysed the percentage change in human mobility intensity at two distinctive points during the COVID‐19 pandemic in Britain. As described in Section 4.1, Figure 3 shows percentage changes in inflows, outflows and intraflows between prepandemic mobility patterns and those after the first lockdown, and after ‘freedom’ day across population density classes. Positive percentage changes indicate increases in mobility intensity relative to the prepandemic period. Reductions in mobility are captured by negative scores and zeros denote no change.

A key pattern from Figure 3 is an overall decline in mobility inflows and outflows across the urban hierarchy after the first national lockdown was enacted. On average, mobility declined by 44% change. The sharpest declines are observed in highly densely populated areas, with declines exceeding 50%. Figure 3 also reveals a high level of variability in inflow and outflow mobility outcomes across the urban hierarchy. While high‐density population areas registered a consistent reduction in mobility intensity, less densely populated areas experienced more variable outcomes. These low‐density areas recorded increases in inflows and outflows, of up to 80% in decile four areas, compared to those mobility levels observed before the pandemic. These areas tend to comprise locales near and around national parks and coastal areas. Instead, reductions in inflows and outflows tended to be larger and concentrate in highly density areas within large cities, including London, Birmingham and Manchester.

Declines in mobility inflows and outflows coincided with an overall rise in intraflows across the urban hierarchy, reflecting the effect of nonpharmaceutical interventions to contain the spread of COVID‐19. Relative to prepandemic levels, intraflow movements increased by an average of 30% (Figure 3). Coupled with business and school closures and working from home, restrictions on outdoor activities and social gatherings foster local mobility to access green spaces and essential services.

Following the full implementation of the government's exit strategy out of the third lockdown in July 2021, we observe increases in mobility intensity across the urban hierarchy (Figure 3). Mobility intensity bounced back closer to prepandemic levels, with less densely populated locations recording levels much closer to prepandemic patterns. Highly dense populous areas continued to record mobility levels between 15% and 20% lower than those occurring before the COVID‐19 pandemic in 2020. Intraflows, however, reported a descending trend across the urban hierarchy. Generally, higher than prepandemic mobility levels are observed in low‐density population areas, gradually declining as population density increases.

### 5.3 Variations in mobility across the urban hierarchy

Based on our modelling estimates, we then examine variations in the intensity of mobility across the urban hierarchy over the course of the pandemic. As described in Section 4.2, estimates were derived from a GLM Quasi‐PRM using the number of moves as a function of a range of variables. Here, we focus our analysis on estimates for our origin‐destination class variables capturing variations in population density across origin and destination pairs. Full regression estimates and model diagnostics are reported in Supporting Information: Figure 3 and Tables 1 and 2.

**Figure 4:**
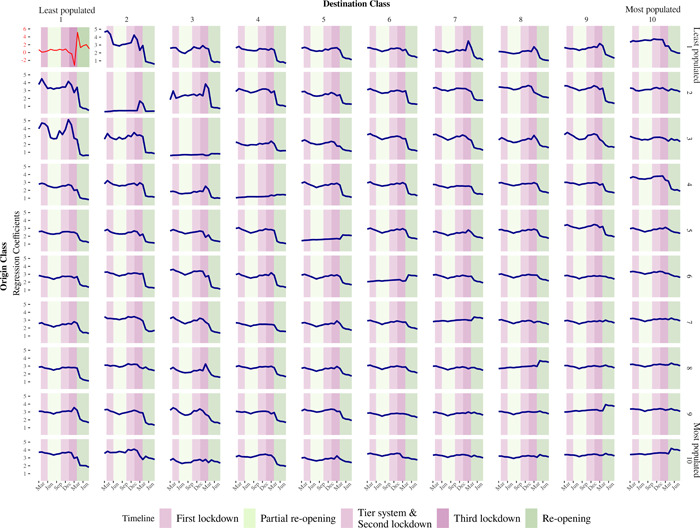
Poisson regression coefficients. The red line represents the regression intercept or reference category from separate monthly regression estimates. The regression intercept plot is reported on a different scale for the reasons discussed in the text. Blue lines represent the regression coefficients for each origin‐destination population density pair, ranging from 1 (the least densely populated areas) to 10 (the most densely populated areas). Confidence Intervals (CIs) based at the 99% level of confidence are shown in grey but are not visible as the regression standard errors are very small.

Figure 4 reports regression estimates for the intercept (i.e., origin and destination Class 1 displaying coefficients with a red line) and origin‐destination class pairs (i.e., all other origin and destination class displayed with blue lines). The *y*‐axis of Figure 4 represents the origin population density classes, while destination classes are reported on the *x*‐axis. Population density classes are encoded with numbers, with 1 indicating the least and 10 denoting the highest population density class. Individual plots are arranged according to these population density classes, with coefficients for the least dense population class at the top left of Figure 4, gradually increasing as we move towards the bottom and right. Each plot displays Poisson regression coefficients (*y*‐axis) for individual origin‐destination population density classes over time (*x*‐axis). These coefficients capture changes in the intensity of mobility between origins and destinations of specific population density levels over the course of the pandemic. Coefficients for origin‐destination pairs were derived from the regression model described in Section 4.2, including a regression intercept as the baseline category. The first plot at the top‐left corner of Figure 4 reports the regression intercept, indicating the expected log mobility count between the least dense origins and destinations if all covariates are zero. This is interpreted as a baseline mobility count estimate, and the set of coefficients reported in the remaining plots are interpreted as deviations from this baseline estimate. Adding the regression intercept and a specific individual origin‐destination pair produces an estimate of the overall effect relating to a particular class. We report individual coefficients for origin‐destination pairs as they enable more easily determining the marginal effect of specific population density class on mobility flows. It enables isolating these marginal effects from overall effects, which capture systematic nation‐wide changes in human mobility patterns. Coefficients for origin‐destination population class pairs indicate the expected log mobility count depending on their density class. A coefficient of 3 for origin Class 10 and destination Class 1, for instance, indicates that an additional 20 (=e(3)) people, on average, moved from the highest to the least population density class, relative to the baseline estimate. For visualisation purposes, we used a different *y*‐axis scale for the regression intercept.

Recalling the national levels of mobility during the period in the analysis is important to contextualise our estimates. The evidence so far (see Figure 3) points to reduced overall levels of mobility after the first national lockdown on 23 March 2020, bouncing back to precoronavirus levels following the implementation of the government's reopening plan out of lockdown starting on 8 March 2021. Focusing first on the regression intercept on the top left of Figure 4, the results show a positive coefficient for the least dense population origins and destinations fluctuating between 0 and 1 from March 2020 to February 2021, with a sharp drop in March 2021 and rise in April 2021, trending to 1 during the period from May to August. These patterns are consistent with low levels of mobility exchanges between the least dense population areas during periods of lockdown and partial reopening during March 2020 to February 2021. They are also consistent with a sudden reduction in mobility between these areas as the first stage of the reopening plan out of lockdown was implemented in 8 March 2021; and, a marked increase as nonessential retail reopen in 12 April 2021.

Figure 4 reveals three key patterns. First, it reveals an overall predominant pattern of relatively high mobility involving low‐density areas (i.e., population density Classes 1–5) during March 2020 to February 2021, rapidly declining during March to August 2021. Figure 4 shows higher than average mobility levels from high, moderate and low‐density population class origin areas (Classes 2–10), particularly to low‐population density class areas (Classes 1–5) during periods of high stringency. We also observe from Figure 4 between origin 1–5 and destination Classes 6–10 higher than average mobility levels from low‐population density class origins to high‐density population destinations. Declining patterns of mobility to and from low‐density population class areas (i.e., population Classes 1–5) post‐February 2021 coincide with the rolling out of the government's four‐staged plan for reopening from March 2021. The extent of this decline displays a clear spatial gradient of large reductions in inflows to low‐population density destinations, moving to more moderate declines in inflows to more densely populated locations. Changes in mobility relating to highly sparsely populated locales stand out displaying relatively high levels of movement, followed by a sudden decline when the third national lockdown was enacted.

A second key pattern relates to population exchanges between highly dense population areas (i.e., population density Classes 6–10) across different population density classes. High‐density destination class areas tend to display relatively stable coefficients between March 2020 and August 2021. This suggests limited variations in the size of population movements in high‐density agglomerations during the pandemic, compared to population exchanges involving low‐density areas.

A third key feature involves population movements between population density areas of similar classes reported in the diagonal of Figure 4. These exchanges tend to show a gradient of low mobility with limited variation in low‐density areas moving to higher levels of mobility in highly dense locations with a sudden rise in intensity following the implementation of the COVID‐19 exit strategy. This sudden increase in mobility intensity seems to have coincided with Stage 2 of the reopening plan involving the resumption of nonessential retail activity and long‐distance travel. These rises reflect increases in mobility exchanges between highly dense areas, but also higher intramobility intensity within these locations.

## 6 DISCUSSION AND CONCLUSION

### 6.1 Key results and interpretation

During the early stages of the COVID‐19 pandemic, anecdotal reports of an ‘urban exodus’ from big cities in various western societies emerged. Using smartphone application data from Facebook users, we sought to analyse the extent and durability of changes in human mobility patterns across the rural–urban hierarchy in Britain during the COVID‐19 pandemic from March 2020 to August 2021. We found evidence of an overall and sustained decline in human mobility between areas during the enactment of nonpharmaceutical interventions between March 2020 and February 2021, bouncing back to precoronavirus levels following the rollout of the government lockdown exit strategy in March 2021. Declines in mobility between areas during high levels of stringency co‐occurred with increases in mobility within areas, probably reflecting rises in active travel and e‐forms of transport (Li et al., 2021). These patterns are largely consistent with evidence emerging from global data from Apple (2020) and Google (2020). We also showed that declines in mobility levels between areas in Britain varied markedly across the urban hierarchy, with the most densely populated areas experiencing the largest reductions; that is, a 60% decline from prepandemic levels. Declines in less populous areas were less acute and more variable, with some areas at intermediate levels of population density displaying rises in population of up to 80% in relation to prepandemic levels. These patterns are consistent with ‘the donut effect’ identified by Ramani and Bloom (2021) to describe a suburbanisation trend of population losses in dense city centres to suburban areas in US cities during COVID‐19.

We presented evidence of higher‐than‐average patterns of mobility from highly dense population areas to low densely populated areas as stringent nonpharmaceutical interventions were enacted and overall levels of mobility declined across Britain. This pattern is consistent with arguments of migration away from dense agglomerations as they became key early epicentres of COVID‐19 infections and lost their urban vibrancy because of business, school closures, social distancing and lockdowns (Florida et al., 2021). International travel restrictions may have also prompted people to stay in Britain and spend more time in rural locations away from dense areas. This is in addition to preexisting housing affordability and poor housing conditions, particularly in cities (Edwards, 2016). Collectively, these challenges seem to have exerted pressure to move out of dense cities as urban life was virtually shut down and small housing units had to be repurposed into multifunctional spaces to accommodate homeschooling, telework and day‐to‐day activities (Capolongo et al., 2020; D'alessandro et al., 2020). However, we also presented evidence of higher‐than‐average mobility in the reverse direction (i.e. from low‐density areas to high‐density locations) and sustained high mobility between areas of relatively high population density. Taken together, our findings indicate that while patterns of population movement from densely populated agglomerations were higher than average reflecting counterurbanisation and suburbanisation processes, we found no evidence of COVID‐19 leading to a ‘population exodus’ from large cities.

We showed evidence of a systematic decline in mobility to low‐density areas, sustained mobility between high‐density areas and a rise in mobility intensity between areas of similarly high population density levels as COVID‐19 restrictions were eased. These findings suggest that while COVID‐19 generated shock waves leading to temporary changes in the patterns of population movement in Britain, the resulting vibrations have not significantly reshaped the prevalent existing structures in the national internal migration system. Large and dense urban areas are likely to remain key centres of population movement. Hybrid forms of working may become widely adopted and predominant ways of interaction. Sparsely rural locations lack the infrastructure and services needed to support hybrid working arrangements (OECD, 2020). Poor broadband connectivity and deficient transport connectivity are likely to represent major challenges for these locations (OECD, 2020). At the same time, urban areas already offer the required digital infrastructure. Economies agglomeration in dense urban spaces are likely to continue to facilitate and foster knowledge exchange, innovation and economic growth (Storper & Venables, 2004). The advent of Internet communication has not led to the geographic dispersal of urban agglomerations (Rietveld & Vickerman 2004), and in a similar way, it is difficult to envisage how the changes brought about by COVID‐19 can trigger an urban exodus and redraw the national pattern of human population settlement.

### 6.2 Limitations and future work

Assessing the wider generalisability of our findings is challenging. Digital footprint data are known to suffer from biases and issues of representation reflecting differences in digital technology penetration, usage and accessibility (Rowe, 2022). In the United States, for instance, young adults aged 20 and 40 tend to be over‐represented in Meta‐Facebook data, while population over the age of 60 appear to be consistently underrepresented (Ribeiro et al., 2020). Yet, these biases do not seem to change our conclusions as these patterns of user representation concur with the mobility age schedule, with high intensity of mobility during young adult ages and gradually decreasing with ageing (Rogers et al., 1978), and given evidence of an increase in internal migration numbers from large cities has been recorded across the age spectrum during COVID‐19 in Spain (González‐Leonardo, Rowe, et al., 2022). Meta‐Facebook data are representative of such age profile predominantly comprising young adult ages. Additionally, a recent UK‐based study using the same mobility Facebook data employed in our study demonstrated that they are strongly correlated with the spatial distribution of census and ONS mid‐year population estimates (Gibbs et al., 2021). It also showed no systematic association between the percentage of Facebook users, and the average age, percent minority ethnic, population density or index of multiple deprivation (Gibbs et al., 2021). Future work could extend our work triangulating other sources of digital footprint data, drawing a larger number of smartphone applications, as well as traditional data sources, like the 2021 British census when it becomes available (Rowe, 2022). Such analysis could provide further ground‐truthing for our results and expand our study distinguishing temporary from more long‐term forms of human mobility such as internal migration.

We analysed the spatial patterns of mobility across the rural–urban continuum. Future work is needed to establish the causes of the observed changes in the spatial direction of mobility during COVID‐19. Understanding these causes can help anticipating long‐term structural changes in mobility intensity extending beyond the pandemic. A combination of factors, including school shutdowns, business closures, social distancing, telework, employment density, housing space and affordability have been cited as key forces altering the preexisting patterns of population movement during the pandemic and triggering moves away from cities. While some of these factors have already dissipated as COVID‐19 restrictions have been lifted, factors such as telework will most likely to endure the pandemic and become the main form of engaging with work (Florida et al., 2021). Assessing the extent to which companies can and will adopt remote work is key to understand the ways in which hybrid working can affect location decisions within and away from cities, to improve and design urban spaces by repurposing office spaces and equip rural locations with needed digital infrastructure and transit connectivity.

## CONFLICT OF INTEREST

The authors declare that there is no conflict of interest.

## ETHICS STATEMENT

The research meets all ethical guidelines, including adherence to the legal requirements of the study country and institutional ethical requirements of the University of Liverpool.

## Supporting information

Supporting information.

## Data Availability

The Facebook data obtained from Meta can be requested via Meta's Data for Good programme: https://dataforgood.facebook.com. Subject to the terms and conditions of a data sharing license agreement, we cannot share or make the data publicly available. The gridded population data were obtained from the Consumer Data Research Centre (CDRC): https://data.cdrc.ac.uk/65304314. Computational code and relevant description to replicate the analysis and results reported in the article can be found in an open‐access Github repository registered on Open Science Framework with DOI: https://doi.org/10.17605/OSF.IO/QHECR.
