# Supplementary material for: Urban exodus? Understanding human mobility in Britain during the COVID‐19 pandemic using Meta‐Facebook data
Source: Popul Space Place. 2022 Dec 7;29(1):e2637. doi: 10.1002/psp.2637 (PMC9877951; doi:10.1002/psp.2637)
Supplement: Supplementary file 1 — Supporting information. [file PSP-29-0-s001.pdf]

Supplementary Material: Urban Exodus? Understanding Human Mobility in Britain During the COVID-19 Pandemic Using Meta-Facebook Data

Prepared for double-blind peer review

Supplementary Tables

Supplementary Table 1. Quasi-Poisson regression coefficients by month, March 2020-August 2021. We report the regression model estimates included in Equation 2, excluding the estimates for origin-destination population density class pairs. Standard errors are provided in brackets.

	Distance	Origin Pop	Destination Pop	Day	Hour 08	Hour 16	Weekend	Quality Score
2020-03	-3.15 (1e-05)	0.179 (2e-06)	-0.037 (2e-06)	-0.146 (2e-06)	-3.859 (3e-06)	0.145 (1e-06)	0.055 (2e-06)	-0.121 (2e-06)
2020-04	0.227 (3e-06)	0.001 (0)	0.16 (2e-06)	-0.054 (0)	0.206 (1e-06)	0 (0)	0.253 (2e-06)	-0.04 (0)
2020-05	0.183 (3e-06)	-0.189 (2e-06)	-0.11 (2e-06)	-3.527 (2e-06)	0.144 (1e-06)	0.066 (2e-06)	-0.125 (2e-06)	-3.304 (2e-06)
2020-06	-0.007 (1e-06)	0.087 (2e-06)	-0.04 (0)	0.22 (1e-06)	-0.003 (0)	0.447 (2e-06)	-0.069 (0)	0.228 (2e-06)
2020-07	-0.211 (4e-06)	-0.043 (2e-06)	-3.724 (2e-06)	0.151 (1e-06)	0.009 (2e-06)	-0.12 (2e-06)	-3.49 (2e-06)	0.102 (2e-06)
2020-08	0.067 (3e-06)	0.064 (0)	0.188 (1e-06)	-0.001 (0)	0.361 (2e-06)	-0.063 (0)	0.231 (2e-06)	-0.002 (0)
2020-09	-0.064 (3e-06)	-3.666 (3e-06)	0.131 (1e-06)	-0.041 (2e-06)	-0.126 (2e-06)	-4.042 (3e-06)	0.107 (2e-06)	-0.019 (2e-06)
2020-10	0.152 (1e-06)	0.234 (1e-06)	0 (0)	0.193 (2e-06)	-0.055 (0)	0.193 (2e-06)	-0.001 (0)	0.216 (2e-06)
2020-11	-3.877 (6e-06)	0.168 (1e-06)	0.042 (2e-06)	-0.102 (2e-06)	-4.197 (3e-06)	0.176 (2e-06)	-0.035 (2e-06)	-0.114 (2e-06)
2020-12	0.233 (2e-06)	0 (0)	0.218 (2e-06)	-0.034 (0)	0.212 (1e-06)	0.009 (0)	0.2 (2e-06)	-0.027 (0)
2021-01	0.191 (2e-06)	-0.152 (2e-06)	-0.189 (2e-06)	-3.711 (3e-06)	0.151 (1e-06)	0.092 (2e-06)	-0.092 (2e-06)	-3.266 (3e-06)
2021-02	0 (0)	0.092 (2e-06)	-0.085 (0)	0.224 (1e-06)	-0.001 (0)	0.447 (2e-06)	-0.01 (0)	0.227 (2e-06)
2021-03	-0.225 (2e-06)	-0.065 (2e-06)	-3.515 (2e-06)	0.154 (1e-06)	0.011 (2e-06)	-0.145 (2e-06)	-3.305 (2e-06)	0.096 (2e-06)
2021-04	0.058 (2e-06)	0.029 (0)	0.21 (1e-06)	-0.001 (0)	0.379 (2e-06)	-0.073 (0)	0.227 (2e-06)	0 (0)
2021-05	-0.048 (2e-06)	-3.727 (3e-06)	0.146 (1e-06)	-0.066 (2e-06)	-0.118 (2e-06)	-3.787 (2e-06)	0.104 (2e-06)	-0.027 (3e-06)
2021-06	0.092 (1e-06)	0.214 (1e-06)	0.001 (0)	0.24 (2e-06)	-0.049 (0)	0.228 (2e-06)	-0.002 (0)	0.23 (3e-06)
2021-07	-3.811 (4e-06)	0.147 (1e-06)	-0.012 (2e-06)	-0.097 (2e-06)	-4.08 (3e-06)	0.129 (2e-06)	0.007 (2e-06)	-0.105 (3e-06)
2021-08	0.236 (2e-06)	-0.003 (0)	0.166 (2e-06)	-0.025 (0)	0.212 (1e-06)	-0.013 (0)	0.215 (2e-06)	-0.037 (1e-06)

Supplementary Table 2. Quasi-Poisson regression model diagnostics by month, March 2020-August 2021. We report model diagnostics for our models, including measures of model fit (i.e. Akaike information criterion, Pseudo R Squared and Log-Likelihood), degrees of freedom and number of observations for each set of monthly estimates.

Month-Year	Akaike	Pseudo R squared	Number of Obs.	Degrees of Freedom	Log-Likelihood
2020-03	5.200832e+11	0.819	291439	291331	-2.600416e+11
2020-04	1.447032e+12	0.826	831840	831732	-7.235162e+11
2020-05	1.714408e+12	0.82	1046802	1046694	-8.572042e+11
2020-06	1.860963e+12	0.809	1227192	1227084	-9.304816e+11
2020-07	1.947047e+12	0.798	1405916	1405808	-9.735233e+11
2020-08	1.822569e+12	0.798	1389682	1389574	-9.112844e+11
2020-09	2.022856e+12	0.79	1462133	1462025	-1.011428e+12
2020-10	2.084085e+12	0.79	1444114	1444006	-1.042042e+12
2020-11	1.930502e+12	0.792	1320533	1320425	-9.652510e+11
2020-12	1.825322e+12	0.796	1296650	1296542	-9.126612e+11
2021-01	1.729239e+12	0.802	1173129	1173021	-8.646193e+11
2021-02	1.596884e+12	0.801	1126290	1126182	-7.984422e+11
2021-03	1.742603e+12	0.823	1290628	1290520	-8.713015e+11
2021-04	1.730555e+12	0.808	1331641	1331533	-8.652774e+11
2021-05	6.719025e+11	0.905	1287563	1287455	-3.359513e+11
2021-06	7.480849e+11	0.9	1498527	1498419	-3.740424e+11
2021-07	7.691850e+11	0.898	1523778	1523670	-3.845925e+11
2021-08	3.720952e+11	0.891	720563	720455	-1.860476e+11

Supplementary Figures

Supplementary Figure 1. Population density classes by decile and adjusted decile classes. As described in Section 3, population density data were classified into ten discrete classes based on deciles. We generated an initial classification which was adjusted to reduce within-population density-class variability and maximise between-population-density-class variability. Specifically, areas belonging to the first four deciles were combined into a single class, the least densely populated category as very little differentiation exists across these classes as shown in the figure below. Areas belonging to the tenth decile were split into four classes based on the tenth decile's quartiles. The figure shows how the adjusted classes which, unlike official classifications (as discussed below), provide a consistent population density classification across the rural-urban continuum, and tend to better capture variations in the distribution of population densities in Britain. Figure 1 shows the spatial distribution of our final population density classification.

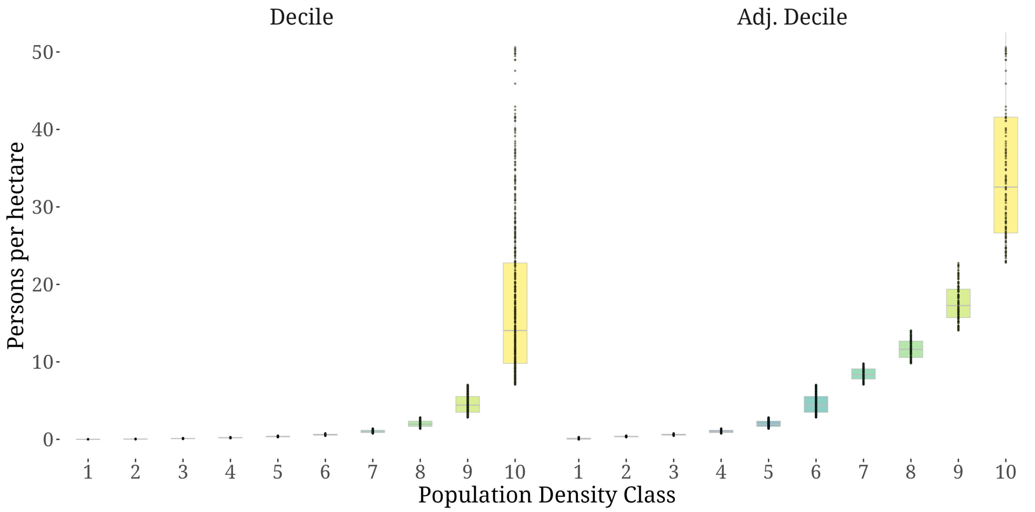

Supplementary Figure 2. Rural-urban classification for Britain. This figure displays the official rural-urban classification covering the British national territory for (a) England and Wales, and (b) for Scotland. These classifications were sourced from the Office for National Statistics and the Scottish Government, respectively. While these classifications offer a categorisation of the national territory across the rural-urban continuum, they are based on different definitions. Hence, we developed our own classification which maps to these categorisations offering a consistent framework.

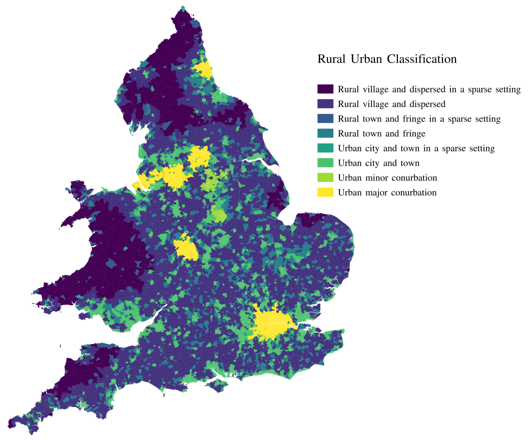

(a) Urban-Rural Classification in England and Wales.

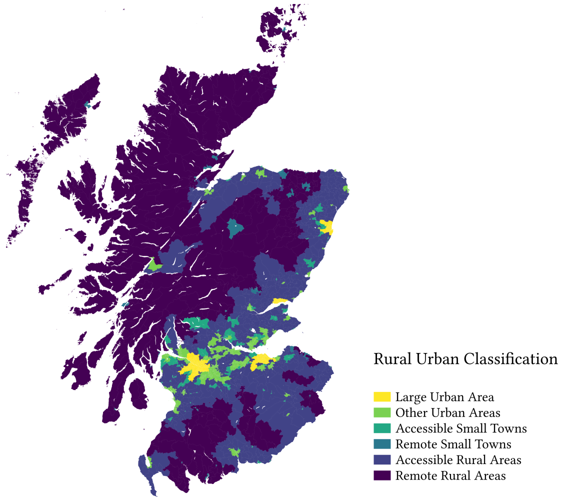

(b) Urban-Rural Classification in Scotland.

Supplementary Figure 3. Quasi-Poisson regression model coefficients by month, March 2020-August 2021. We plot the regression model estimates included in Equation 2, excluding the estimates for origin-destination population density class pairs. These estimates are reported in Figure 4. Detailed information on our model specification estimation can be found in Section 4.2. In the figure below, four colours are used to differentiate between model components: 1) distance; 2) population size at the origin and destination (i.e. OD population); 3) quality score, which is a metric provided by Facebook assessing the uncertainty in mobility flow estimates; and, 4) time, which refers to the temporal dynamics of our model involving a eight-hour window, day trend, and differences between weekdays and weekends.

The figure below shows a remarkably consistent association between time, OD population and quality score components and mobility flows over the course of COVID-19 pandemic, with coefficients ranging from -0.2 to 0.4. All coefficients show the expected direction of influence with larger populations at origin and destination, relating larger population movements. Coefficients for day, hours and weekend reflect small variations in the size of mobility flows over time, reflecting time trend linear changes, fluctuations in during the course of the day, and between weekdays and weekends.

As expected, distance shows a statistically significant negative coefficient, pointing to a negative association between the size of mobility flows and geographical distance. There is some variation in this association over time which tends to coincide with changes in stringency. Periods of high levels of stringency tend to correlate with greater coefficients for distance, reflecting the fact that people were deterred from moving over long distances and encourage to move within the local area.

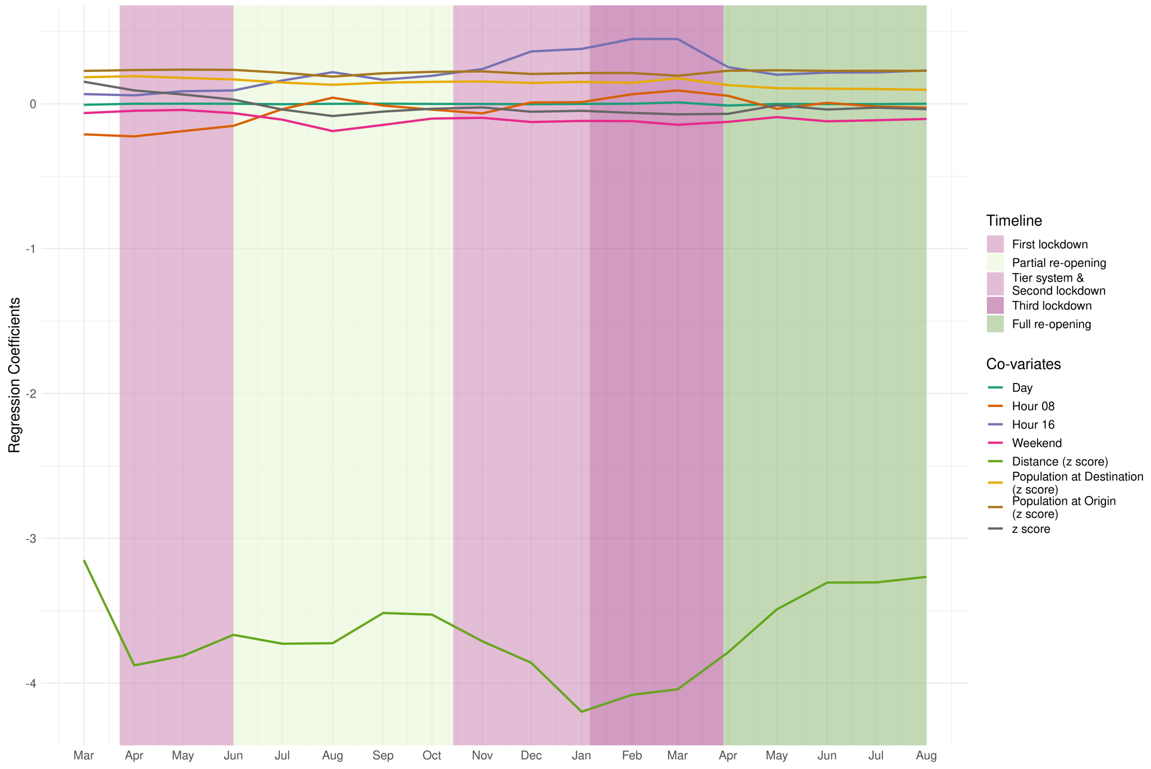
Regression Coefficients
0
-1
-2
-3
-4
Mar
Apr
May
Jun
Jul
Aug
Sep
Oct
Nov
Dec
Jan
Feb
Mar
Apr
May
Jun
Jul
Aug
Timeline
First lockdown
Partial re-opening
Tier system & Second lockdown
Third lockdown
Full re-opening
Co-variates
Day
Hour 08
Hour 16
Weekend
Distance (z score)
Population at Destination (z score)
Population at Origin (z score)
z score
